# Combined Ultrasound Imaging and Biomechanical Modeling to Estimate Triceps Brachii Musculotendon Changes in Stroke Survivors

**DOI:** 10.1155/2016/5275768

**Published:** 2016-12-08

**Authors:** Le Li, Raymond Kai-yu Tong

**Affiliations:** ^1^Department of Rehabilitation Medicine, The First Affiliated Hospital, Sun Yat-sen University, Guangzhou, China; ^2^Department of Physical Medicine and Rehabilitation, University of Texas Health Science Center at Houston and Research Center at TIRR Memorial Hermann Hospital, Houston, TX, USA; ^3^Biomedical Engineering Division, Department of Electronic Engineering, The Chinese University of Hong Kong, Sha Tin, Hong Kong

## Abstract

The aim of this study was to investigate the changes of musculotendon parameters of triceps brachii in persons after stroke based on subject-specific biomechanical modeling technique combined with in vivo ultrasound measurement. Five chronic stroke survivors and five normal control subjects were recruited. B-mode ultrasound was applied to measure muscle pennation angle and the optimal length of three heads of triceps' brachii at different joint angle positions in resting and isometric contraction. Measured ultrasound data were used to reduce the unknown parameters during the modeling optimization process. The results showed that pennation angles varied with joint angles, and the longhead TRI pennation from stroke group was smaller than the literature value. The maximum isometric muscle stress from persons after stroke was significantly smaller than that found in the unimpaired subjects. The prediction of joint torque fits well with the measured data from the control group, whereas the prediction error is larger in results from persons after stroke. In vivo parameters from ultrasound data could help to build a subject-specific biomechanical model of elbow extensor for both unimpaired and hemiplegic subjects, and then the results driven from the model could enhance the understanding of motor function changes for persons after stroke.

## 1. Introduction

The movement disorders presented in persons after stroke include weakness, spasticity, and muscle cocontraction, which causes difficulties for achieving critical activities in daily life [1–3]. Coordinated human movement is a complex behavior, even for a seemingly simple one-degree-of-freedom task. For example, elbow extension movement is modulated by the coordinated action of at least three extensor muscles [4]. Forces generated by these muscles are transferred to the bones via the tendons and affect motion of the joint [5]. Since in vivo muscle forces cannot be measured directly, computer modeling is a useful tool for enhancing our understanding of muscle force change related to aging [6] and pathological disease [7, 8]. The modeling approach can describe the kinetic response of activated muscle at different angle contractions, represent the force producing characteristics of the muscle, and compute the individual muscle force and moment during motor tasks. The modeling technique has been successfully applied to many human joints of the upper arm, such as the hand [9], wrist [10, 11], and elbow [7, 12–14].

However, one of the major challenges in musculoskeletal modeling is to accurately estimate the musculotendon parameters on a subject-specific basis. Sensitivity analysis has shown that musculoskeletal model behavior tends to be very sensitive to the values of musculotendon parameters [11, 15]. For example, when a muscle is constantly activated, its moment arm, physiological cross section area (PCSA), and its operating range (what portion of the isometric force-length relationship curve the muscles use during joint rotation) are the key factors that characterize maximum moment-generating capacity as a function of joint position [16].

Ultrasound imaging techniques have been used to obtain the parameters of musculotendon in vivo and noninvasively [17]. Accuracy of the ultrasound method in measuring muscle architecture features has also been demonstrated to show good agreement with direct anatomical measurement on a cadaver [18]. The pennation angle and muscle fascicle length are two architectural variables readily measured by using ultrasound imaging previously [19, 20]. Our previous ultrasound study on brachialis of persons after stroke showed there were significant muscle changes of affected side compared to those from the unaffected side [21]. Stroke survivors often present a flexed elbow [22]. The literature has shown that elbow extension impairment after stroke is sometimes even worse than flexion. For example, Zackowski and colleagues investigated reaching movement from hemiparetic subjects and found reaching out movement which involved more elbow extension was worse versus the reaching up condition [3]. In addition, in a review of clinical studies of upper movement in hemiplegic cerebral palsy, the results had shown that elbow extension is reduced and compensated by increased truck flexion to reach an object [23]. However, there is limited knowledge about the architectural changes of triceps brachii after stroke as well as how these muscle structural alterations contribute to the changes of force generation in stroke survivors. Therefore, the investigation of the mechanisms underlying changes in elbow extension after neurological insult is warranted, and biological modeling could provide important insights into it. Due to the variation of the impairments on persons after stroke, it may be better to obtain the parameters in vivo to acquire more precise information for the modeling on the specific subjects.

This study extended our previous efforts on elbow flexor modeling to design a subject-specific method of musculoskeletal model using in vivo ultrasound data on the elbow extensors and aimed to investigate the feasibility of estimating the musculotendon parameters during elbow extensions of both unimpaired subjects and persons after stroke. Our hypothesis is that the maximal isometric muscle stress, one of the modeling outputs, would be less on the affected side of stroke group compared to that from control group. In addition, different contributions of three heads of triceps (long, lateral, and medial) to elbow extension could also be evaluated and compared between stroke and control subjects. It is hoped that, through this ultrasound-combined subject-specific technique, we can enhance our understanding of the muscle parameters and force generating capacity changes of individual stroke survivors on elbow extension and help to design suitable rehabilitation intervention for them.

## 2. Materials and Methods

### 2.1. Subject Recruitment

Five persons after stroke and five normal subjects were recruited for this study. Clinical characteristics of the persons after stroke were summarized in Table 1. The selection criteria were (1) a single unilateral lesion of the brain with the duration of stroke more than 1 year; (2) having spasticity with Modified Ashworth Score (MAS) larger than 1 and sufficient passive range of motion (flexion is up to 105°) at the elbow joint; (3) adequate mental ability to understand the experimental tasks as instructed; (4) no surgical procedure on the affected side of the upper limb; (5) absence of significant medical complications. The participants gave informed consent following the ethical procedures and this study was approved by the human subject ethics subcommittee of local university.

### 2.2. Model Consideration

In this study, cylinder and sphere shape of wrapping objects have been applied to simulate the humerus head and capitulum on the muscle path in the geometry model. Via points were determined to allow the muscle to move around the bone surface during joint movement. The whole wrapping muscle path was then defined by connecting the origin, via points, and insertion point of that particular muscle [25]. The musculotendon length (*l*_*mt*_) from muscle origin to muscle insertion points was estimated through summing each line segment on the whole muscle path in SIMM (Software for Interactive Musculoskeletal Modeling, MusculoGraphics, Inc., USA) and then scaled on subject-specific bases based on the segment ratio from anthropometric measurements. Then, *l*_*mt*_ was determined as follows:

(1) $$\begin{array}{c} l_{mt}=\overset{n-1}{\underset{i=1}{\sum }}\left|\;P_{i+1}-P_{i}\;\right|, \end{array}$$

where *P*_1_, *P*_2_,…, *P*_*n*_ are muscle via and attachment points on the path of each muscle and we allowed the joint angle range of 0°–130° with an increment of 1°.

*l*_*mt*_ is then used to estimate moment arm (MA) as the following partial derivative of joint angle (*θ*) with the following equation:

(2) $$\begin{array}{c} \text{MA}=\frac{\partial l_{mt}}{\partial \theta }. \end{array}$$

The biomechanical characteristics of musculotendon dynamics could be evaluated by a modified Hill-type model that describes force-length and force-velocity relationships of the muscle at active and passive contraction conditions and the elastic properties of the tendon [26]. The force generated by each musculotendon complex can be calculated as in the following equations:

(3) Ft=Fm·cos⁡α=Fzfalfvat+fplcos⁡α,

(4) Fz=PCSA·σm,

where *F*_*z*_ is the maximum isometric muscle force, *σ*_*m*_ is the maximum isometric muscle stress, *a*(*t*) is the activation level, and *α* is the pennation angle. During maximum isometric voluntary elbow extension, it is assumed that all the elbow extensors are fully activated (i.e., *a*(*t*) = 1). *f*_*a*_(*l*) is the force-length relationship of contractile element and *f*_*p*_(*l*) is the parallel muscle force of passive elastic element [27]:

(5) fpl=AP·ekpel−lmo/lmo−1,fal=sinb1·llmo2+b2·llmo+b3.

Herein, the coefficients *A*_*P*_ = 0.129, *k*_*pe*_ = 4.525, *b*_1_ = −1.317, *b*_2_ = −0.403, and *b*_3_ = 2.454.

Tendon is taken as a nonlinear spring for which the nominal force-strain relationship is composed of two regions (i.e., an initial exponential relationship and a linear relationship for larger deformations) which satisfied the following relationship:

(6) $$\begin{array}{c} F_{t}\left(\;\varepsilon_{t}\;\right)=\left\{\begin{array}{cc} F_{z}\cdot A\cdot \left(e^{k_{1}\varepsilon_{t}}-1\right) & 0\leq \varepsilon_{t}<\varepsilon_{c}\\ k_{2}\cdot F_{z}\cdot \left(\varepsilon_{t}-\varepsilon_{c}\right)+F_{c} & \varepsilon_{t}\geq \varepsilon_{c}, \end{array}\right. \end{array}$$

where *ε*_*t*_ is tendon strain at tendon length *l*_*t*_ (i.e., *ε*_*t*_ = (*l*_*t*_ − *l*_*to*_)/*l*_*to*_), *ε*_*c*_ is the tendon strain at tendon length *l*_*tc*_ (i.e., *ε*_*c*_ = (*l*_*tc*_ − *l*_*to*_)/*l*_*to*_), *l*_*to*_ is the tendon slack length, and *l*_*tc*_ is the tendon length at which tendon force shifts from nonlinear to linear region. Based on the experimental results from other studies, Zajac [26] constructed a generic nominal force-strain curve of tendon and estimated the dimensionless coefficients for the tendon force-strain relationship: *A* = 0.1238; *k*_1_ = 81.1438; *k*_2_ = 37.5; and *F*_*c*_/*F*_*z*_ = 0.5. In addition, Zajac [26] found the linear region that begins after tendon is stretched by 2% (i.e., *ε*_*c*_ = 0.02) and the corresponding stress is 16 MPa; the strain of the tendon at which the tendon force equals maximum isometric muscle force *F*_*z*_ is tendon independent and equals 3.3% and the corresponding stress is also tendon independent and equals 32 MPa [26].

Equation (6) is used to calculate tendon force, and together with (3)~(5) on estimating muscle force, all the equations are substituted into (3).

Tendon length (*l*_*t*_) was estimated from the whole musculotendon length (*l*_*mt*_), muscle fascicle length (*l*_*m*_), and pennation angle (*α*):

(7) $$\begin{array}{c} l_{t}=l_{mt}-l_{m}\cos\alpha . \end{array}$$

The torque generated by the elbow extensors during maximum voluntary contraction (MVC) could be calculated from the summation of each muscle's contribution, considering the moment arm of each muscle:

(8) $$\begin{array}{c} T\left(\;\theta \;\right)=\overset{3}{\underset{i=1}{\sum }}F_{i}\left(\;\theta \;\right)\times {\text{MA}}_{i}\left(\;\theta \;\right), \end{array}$$

where *F*_*i*_(*θ*) is the tendon force and MA_*i*_(*θ*) is the moment arm of each prime elbow extensor *i* (i.e., 1: the medial head of triceps brachi (MHT), 2: the lateral head of triceps brachii (LatHT), and 3: long head of triceps brachii (LngHT) at joint position *θ*).

### 2.3. Ultrasound Measurement

B-mode ultrasound machine with a 7.5-MHz, 38 mm probe (resolution of 0.3 mm) (Sonosite 180 Plux, Sonosite Inc., USA) was applied to measure elbow extensor's pennation angle and optimal length at the optimal angle when the muscle is fully activated [28]. Based on (7) and the definitions of muscle optimal length and tendon slack length, the following equation was applied for the calculation of the tendon slack length (*l*_*to*_):

(9) $$\begin{array}{c} l_{to}=l_{mt}^{o}-l_{mo}\cdot \cos\alpha_{o}, \end{array}$$

where *l*_*mt*_^*o*^, *l*_*mo*_, and *α*_*o*_ are the musculotendon length, muscle optimal length, and pennation angle at the optimal angle of the elbow joint, respectively.

The optimal angle, which is the joint angle that corresponds to the muscle optimal length, determines the operating range in a length-tension relationship by the joint movement and tendon excursion. Previous studies reported the optimal joint angle of elbow extension is 90° [29] and since the triceps brachii contribute nearly 80% of the moment to the joint, it is reasonable to assume that the 90° was set as optimal angle of triceps in this study. Similar angle was applied in a study in literature recently [16].

During the experiment, the subject was seated in a height-adjustable chair, with the arm to be tested put on the arm holder of a custom-made dynamometer (motor: Dynaserv, Yokogawa, Japan; torque sensor: AKC-205A, China Academy of Aerospace Aerodynamics, accuracy of 0.03 Nm, China). The motor can drive the arm holder to exact testing position and the torque sensor could measure the generated elbow extension torque from the subject during isometric contraction. The testing plane of the arm is parallel to the floor at the same height as the shoulder, with the shoulder abducted 90° and flexed 0°.

**Figure 1:**
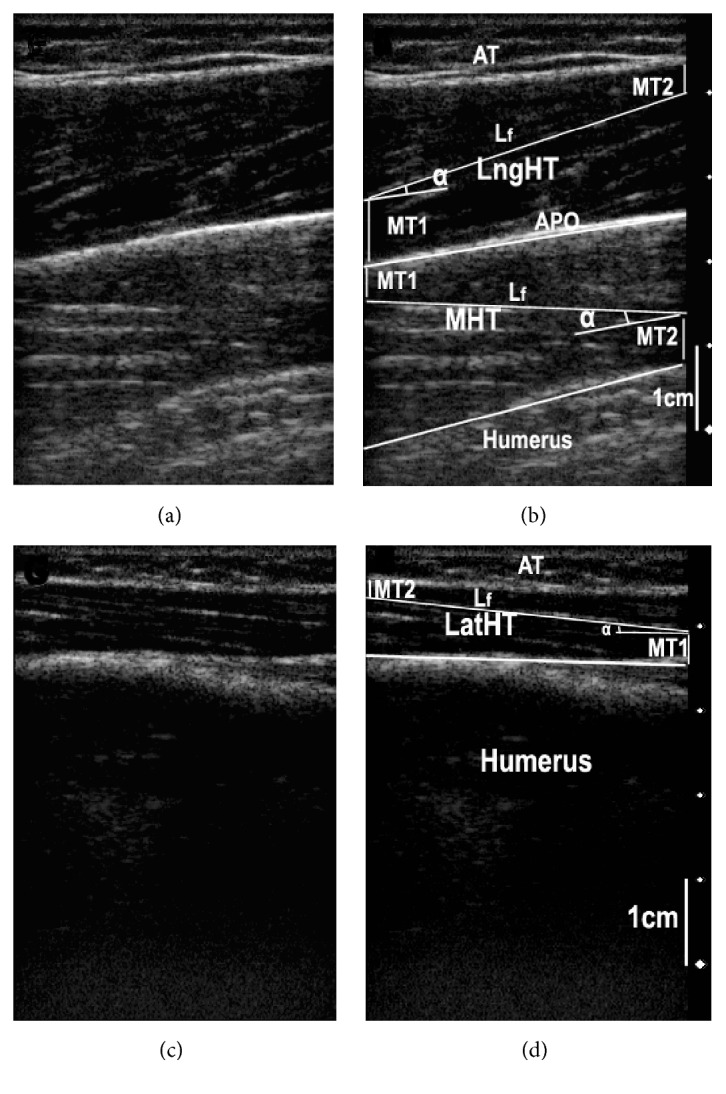
Typical ultrasonography imaging of the prime elbow extensors: long head of triceps brachii (LngHT), medial head of triceps brachii (MHT), and lateral head of triceps brachii muscle. (a) Original ultrasound image of MHT and LngHT; (b) MHT and LngHT image with labels; (c) original ultrasound image of LatHT; and (d) LatHT image with labels. In (a) and (b), the APO (bright fringe in the middle region) shows the boundary between MHT and LngHT. The white fringe in the lower range is the muscle-bone boundary (MHT-humerus). In (c) and (d), the bright fringe shows the muscle-bone boundary (LatHT-humerus). In (b) and (d), *L*_*f*_ is the visualized part of the entire muscle fascicle length; MT_1 _and MT_2_ are the distance of the fiber proximal end to the bone and the distance of the fiber distal end point to the superficial aponeurosis, respectively; AT is adipose tissue; *α* is the pennation angle.

For each elbow joint position from 0° to 105° with 15° increment, the probe was put in the posterior part of the upper arm and the position of the probe was on the muscle belly which is just in the middle of the upper arm, for measuring MHT and LngHT. The probe was then parallelly moved 2 cm to the lateral direction to measure LatHT. Coupling gel (Parker Aquasonic 100 Gel, USA) was applied to enhance ultrasound conduction between the ultrasound probe and the skin surface. Typical ultrasound images taken of the prime elbow extensors and the demonstration of calculating the muscle architectural parameters are shown in Figure 1. The ultrasound images were stored in the computer and analyzed offline (UTHSCSA imaging tool, USA) to estimate the muscle architecture parameters.

### 2.4. Torque and EMG Data Recording

**Figure 2:**
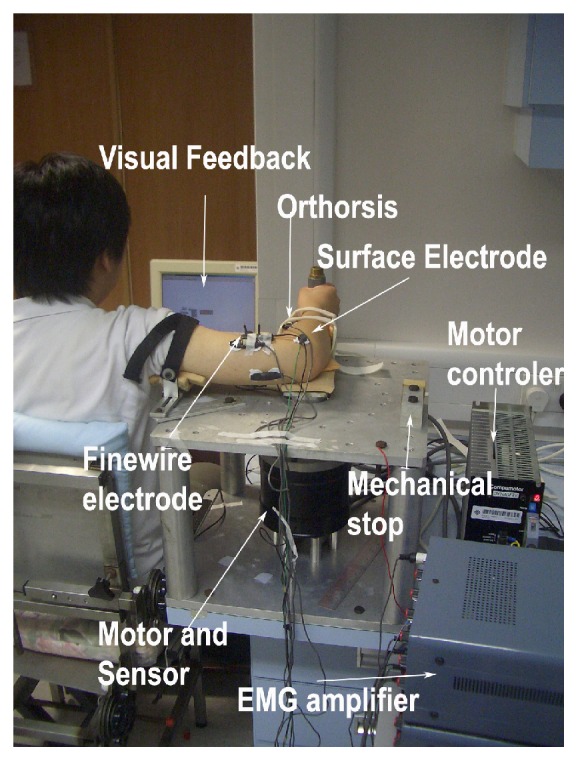
Experiment setup of EMG and torque data collection in MVC. Surface EMG electrodes were attached on long, medial, and lateral head of triceps brachii. In this photo, the elbow was positioned at the 90° flexed position.

The setup of experiment in second stage of EMG and torque data collection was shown in Figure 2. The testing position of torque and EMG is similar to ultrasound measurement. Briefly, each subject sat on the assessment chair and the forearm was attached to the arm holder, which was connected to the end of the torque sensor. An orthosis with semicircular cross section was attached to the arm holder. The subject's forearm was placed inside the orthosis and straps were used to fasten the forearm. The upper arm was also fastened by a strap to a supporter mounted on the upper aluminum plate. The orthosis and arm holder could guide the forearm to rotate with an axis of rotation in line with the motor and the torque sensor. The subject was asked to grasp the handle of the arm holder and could voluntarily perform elbow extension in the horizontal plane. A screen with visual feedback was placed in front of the subject to provide guidance (Figure 2). There were three trials of extension at each testing position and each contraction lasted for around 5 seconds. A two-minute recovery period was allowed between contractions to minimize muscle fatigue.

In this study, surface electrodes were applied to record the myoelectric activities of the target muscles to record the activities of many motor units within a muscle [30]. Bipolar pregelled Ag/AgCl surface electrodes (Noraxon dual electrode, Noraxon Inc., USA), placed 3 cm apart, were used to record EMG of the three elbow extensors. An additional reference electrode was placed distant lateral of the elbow, over the skin surface of olecranon. Electrode placement was verified by functional muscle testing. Torque and EMG signals were recorded with a sampling frequency of 2000 Hz and stored on a PC computer via the data acquisition (DAQ) card (PCI 6036E, National Instrument, Texas, USA). The amplifier gain for surface EMG signal is 1000.

### 2.5. EMG and Torque Data Processing

The digitized EMG and elbow torque signals were processed offline. A detailed analysis on MVC torques and the associated EMG of prime elbow extensors was performed. The torque signals were low-pass filtered (fourth-order Butterworth filter) at 5 Hz. The torque data measured at different elbow angles were curved-fitted to the lowest-order polynomial equation with an* R* ^2^ value greater than 0.9. This torque-angle relationship would be incorporated into the model to calculate the musculotendon parameters of each target muscle. The raw surface EMG were band-pass filtered (fourth-order Butterworth filter 10 Hz–500 Hz) and then rectified and further processed with a 100 ms moving average window. The filtered EMG signals were normalized by peak value of EMG amplitude measured during the MVC test.

### 2.6. Optimization Process

The optimization program calculated the individual maximum muscle force for each prime elbow extensor individually. The optimization algorithm tried to minimize the root mean square difference between the polynomial fitted with a third-order function and torque-sensor measured maximal isometric extension torques with the range from 10° to 100° at elbow joint, with increments of 2° [31]:

(10) $$\begin{array}{c} \text{minimize }F=\sqrt{\frac{\sum_{i=1}^{46}{\left(T_{i}^{m}-T_{i}^{p}\right)}^{2}}{46}}, \end{array}$$

where *T* ^*m*^ is the measured elbow extension torque and *T* ^*p*^ is the predicted elbow extension torque. As mentioned before, the input parameters of the model included the muscle optimal length and pennation angle from the ultrasound measurements and the musculotendon length and moment arm from the geometric model after subject-specific scaling based on segment length from upper arm and forearm. The optimization of maximum isometric muscle force was conducted in two steps. In the first step, the optimization scheme would produce the value of maximum isometric muscle stress in order to obtain a suitable initial value and proper searching boundary for the main process of the optimization in the second step. In step 1, the same maximum isometric muscle stress value was assumed for all prime elbow extensors [13]. The value of PCSA for each muscle was scaled using literature data [32], based on the ratio of the upper arm circumference of cadaver and our subjects. The search process was one full dimensional Nelder-based simplex call [33] and it continued until the root mean square difference between the measured torque and the estimated torque was minimized. The results from the first step provided the value of maximum isometric muscle stress of the prime elbow extensors which was multiplied by PCSA of each muscle to obtain the initial value of maximum isometric muscle force to be used in the second step of optimization. In step 2, plus and minus 15% percent of the initial value were used as the upper and lower boundaries (for the stroke simulation, the initial boundary was set to 30% considering the lower activation level of the affected muscle due to the stroke). The three muscles' maximum isometric muscle forces became variables and the same optimization scheme and constraints as the first step were applied in the second step. The optimization scheme was to stop when the root mean square difference of the measured torque and calculated torque was minimized. After obtaining the maximum isometric muscle force, the maximum isometric muscle stress was calculated by dividing the force by the corresponding PCSA of each muscle [32].

### 2.7. Statistical Analysis

One-way repeated measures analysis of variance (ANOVA) was applied to evaluate if there are significant differences of the pennation angle of each muscle in different elbow joint positions. Linear regression was used to describe the relationship with ultrasound-measured muscle pennation angle and the joint angle. One-way ANOVA with post hoc Bonferroni test was also used to evaluate optimal muscle length, tendon slack length, and maximum isometric muscle stress (*σ*_*m*_) from different elbow extensor muscles in each subject group. If there is no significance, data from three muscles would be combined together and* t*-tests were used to compare the group difference between persons after stroke and health control.* p* values less than 0.05 were regarded as statistical significance.

## 3. Results

**Figure 3:**
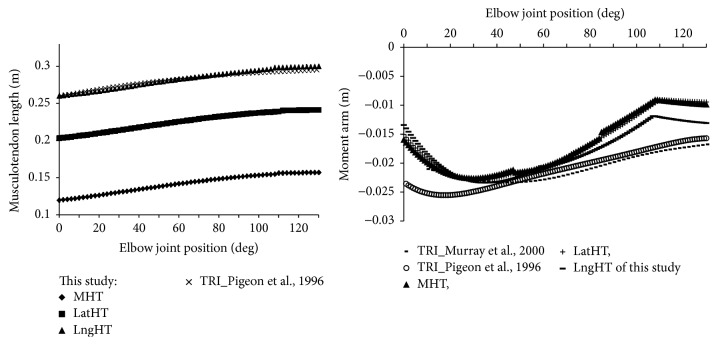
Musculotendon length (a) and moment arm (b) of elbow prime extensors of this study, that is, medial head of triceps (MHT), lateral head of triceps (LatHT), and long head of triceps (LngHT) of this study, compared with corresponding literature results from Pigeon et al. [34] and Murray et al. [32]. Pigeon et al. [34] derived data in other studies to obtain a relationship between joint angles and upper limb muscle parameters by curve fitting. MA data from Murray et al.'s study was based on the polynomial equations and the linear regression equations between anthropometric variables and peak moment arm derived by Murray et al. [32].

Figure 3(a) shows the musculotendon length of each prime elbow extensor in this study from a typical subject (CO, male in Table 1) and musculotendon length of triceps brachii muscle as a whole from literature [34]. *l*_*mt*_ from our estimation had similar trend to that of the literature that *l*_*mt*_ of elbow extensor increased about 21% as the joint angle flexed from extended position (0°) to flexed position (130°). The moment arm of three prime elbow extensors estimated from geometric model was shown in Figure 3(b). The moment arm-angle results for the range of 0°–130° with an increment of 1° were compared with literature cadaver data [34, 35]. The average moment arm of each muscle over the range of 0°–130° was as follows: MHT = 1.70 cm; LatHT = 1.71 cm; LngHT = 1.81 cm.

**Figure 4:**
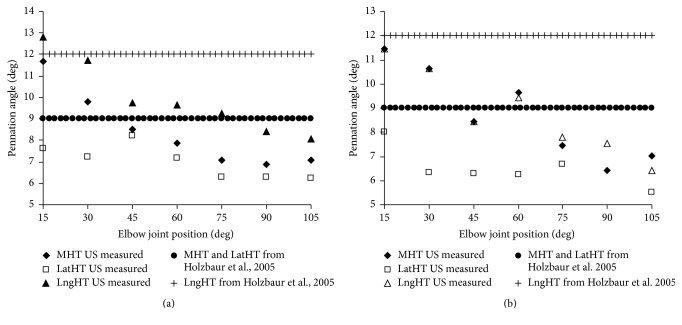
In vivo measurement of pennation angles of elbow extensors to the joint angle in the MVC condition for unimpaired group (a) and hemiparetic group (b) as well as pennation angle from literature [36].

Figure 4 shows the ultrasound-measured pennation angles (mean values) of MHT, LatHT, and LngHT of unimpaired group (Figure 4(a)) and hemiparetic group (Figure 4(b)), as well as pennation angles adopted in literature [36]. The results revealed that the in vivo pennation angle of elbow extensor was angle-depended with difference of measure joint position (one-way ANOVA, *p* < 0.05) in both unimpaired and hemiparetic group. For unimpaired group, the pennation angles decreased from 11.7° to 7.1°, 7.6° to 6.3°, and 12.8° to 8.1° for MHT, LatHT, and LngHT, respectively, as the joint position changed from 15° to 105°. The linear regression results from unimpaired group showed MHT (*α* = −0.05*θ* + 11.43;* R* ^2^ = 0.843) and LngHT (*α* = −0.051*θ* + 12.99;* R* ^2^ = 0.913) have similar slope and initial value which, both of them, were larger than those from LatHT (*α* = 0.019*θ* + 8.14;* R* ^2^ = 0.648). Similar pennation angle from hemiparetic group was observed when compared with unimpaired group.

**Figure 5:**
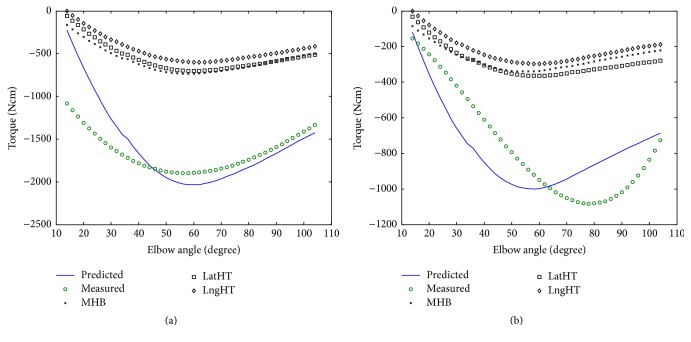
Modeling results: comparison of typical predicted profiles with measured extension torque-angle results for (a) one unimpaired subject and (b) one person after stroke. Individual elbow extensor generated torque-angle profiles were also plotted.

Figure 5 shows the typical result of the measured and predicted maximum isometric extension torque versus elbow joint angle for a subject in unimpaired group (a) and for one hemiparetic subject (b). In general, the predicted torque-angle fits the measured one well except at the more extended positions (<50°). The RMS differences ranged from 1.43 to 6.78 Nm (mean = 3.64, SD = 2.25, *n* = 5). The predicted torque-angle profile follows the similar trend as the measured one with the RMS differences ranging from 1.76 to 4.33 Nm (mean = 3.01, SD = 0.95, *n* = 5) for extension in hemiplegic group.

Muscle optimal lengths of the three heads of triceps (MHT, LatHT, and LngHT) in the unimpaired group were found to have no significant difference (*p* = 0.34) among them and with a mean and SD of 8.8 ± 2.2, 11.0 ± 2.8, and 9.2 ± 2.3 cm, respectively (Table 2). The mean and SD of tendon slack lengths were 5.7 ± 1.3, 11.4 ± 1.9, and 18.7 ± 1.2 cm, respectively, and were found to be significantly different (*p* < 0.001) in the unimpaired group. Post hoc results showed *l*_*to*_ from MHT was significantly smaller (*p* < 0.001) than that from LatHT and also significantly smaller (*p* < 0.001) than that from LngHT. Similarly, the mean muscle optimal lengths of the three heads of triceps (MHT, LatHT, and LngHT) in hemiplegic group were found to be not significantly different (*p* = 0.241) with a mean and SD of 10.9 ± 2.1, 12.8 ± 1.9, and 10.5 ± 2.4 cm, respectively. The mean and SD of tendon slack lengths were 3.2 ± 1.6, 9.3 ± 2.7, and 17.0 ± 2.3 cm, respectively, and were found to be significantly different among them (*p* < 0.001). Post hoc results showed *l*_*to*_ from MHT was significantly smaller (*p* = 0.003) than that from LatHT and also significantly smaller (*p* < 0.001) than that from LngHT in hemiparetic group.

**Figure 6:**
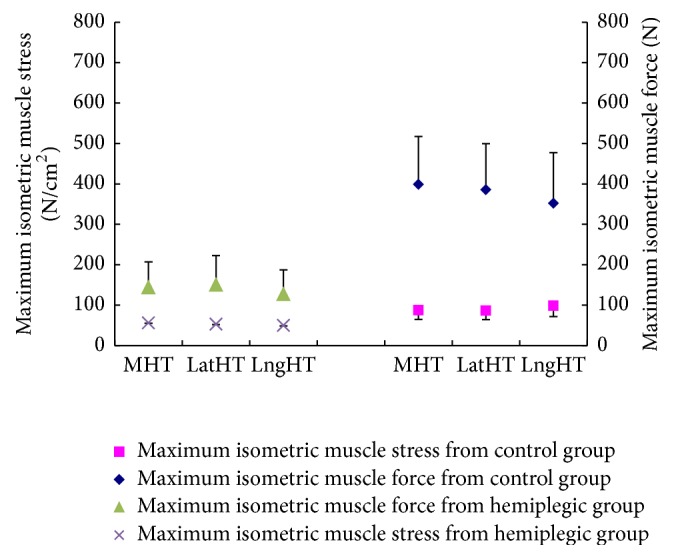
Maximum isometric muscle force and maximum muscle stress (mean value, *n* = 5) for each prime elbow extensor for unimpaired group and hemiparetic group. The error bar indicates one-standard-deviation length.

Figure 6 shows there was no significant difference in *σ*_*m*_ (maximum isometric muscle stress) of LatHT, LngHT, and MHT in hemiparetic group and unimpaired group, respectively. Therefore, the mean value (*σ*_*e*_) of these three prime elbow extensors was used in comparisons below. The value of maximum isometric muscle stress for extensor of unimpaired group was found to range from 60.9 to 115.4 N/cm^2^. In hemiparetic group, the maximum muscle stress of extensor group was found to range from 18.5 N/cm^2^ to 66.1 N/cm^2^. The mean ± SD for the unimpaired and hemiparetic group was 89.3 ± 21.8 and 50.8 ± 18.6 N/cm^2^, respectively, and the value from hemiparetic group was found to be significantly smaller than that of the unimpaired group (*p* = 0.023).

## 4. Discussion

In this study, a biomechanical model of elbow extensors was built for both unimpaired subjects and persons after stroke based on subject-specific ultrasound-measured and experimentally optimized parameters.

### 4.1. Muscle Path and Moment Arm

The results showed that *l*_*mt*_ in this study of LngHT is almost the same as literature *l*_*mt*_ of triceps brachii [18]. In general, the modeled moment arms matched well with the data from the literature in terms of the trend and amplitude. Similar moment arms were noted among the three heads of triceps and discontinuities in the moment arm were noted in flexed position about 110° where the additional via points become active.

Previous literature outlined methods of calculating muscle moment arm based on consideration of the line of action or tendon excursion [35, 37]. Furthermore, in other studies, muscle length was defined as linear functions [38] or nonlinear functions of joint angles [34] or a straight line from origin to insertion, which might not represent accurately the real complex morphology of passive structures such as joint articulating surfaces and ligaments. Another study used the constraint points of the muscle path and defined them using interpolation point technology [13]. However, doing this probably resulted in the muscle “punching through” the bone when the joint angle was changed to an extreme position. This is unreasonable and may lead to the calculated moment arm being disconnected. In addition, the constraint points needed to be defined individually on each degree of freedom (DOF), which becomes a very complex task when multi-DOF movement is to be modeled. In one of the modeling studies, the investigators used geometric-shaped objects to make the muscle wrap its path smoothly over bony landmarks or other constraints [39]. The wrapping objects could constrain via points of the muscles moving on the bone contours. In our study, cylindrical and spherical shapes for wrapping objects have been applied to simulate the humerus head and capitulum on the muscle path and then have effects on the moment arm. The general profile of the moment arm across the joint positions was similar to the literature but our calculated moment arm was continuous, making the later calculating of joint moment more reliable. It is difficult to access the accuracy of moment arm directly. It was believed that the discrepancy might be due to the underestimation of the triceps moment arms at the more extended positions. Another reason for the discrepancy may be the optimal angle selection since this value was observed from the active contraction. The accuracy of musculotendon length and moment arm is important to the later estimation of muscle force and joint trajectory [40]. The results of the geometric model, such as musculotendon length and moment arm, could be used further for 3D motion analysis or force prediction in dynamic conditions [30].

### 4.2. Musculotendon Parameters and Ultrasound Measurement

Appropriate musculoskeletal modeling can provide both qualitative and quantitative information into the neuromusculoskeletal system and its motion dynamics to analyze human movement [4]. The modeling parameters are important to the success of the model. Manal and Buchanan (2004) used a numerical method based on Hill-type model to estimate the elbow muscle architecture parameters and they found that the 5% change of the tendon slack length resulted in upwards 30% difference on the estimated output fiber force [5]. Especially in poststroke survivors, the muscle parameters have changed after the insult [14]. In this current study, the pennation of lateral head of triceps of this study was smaller (about 9°) than that from the literature [36]. The results also showed that the in vivo pennation angle of long head of triceps in hemiparetic group was smaller (around 12°) than the literature value. In literature, it is suggested better to obtain subject-specific modeling parameters to tailed individual biomechanical modeling. For example, Hasson and Caldwell found subject-specific models in aging study gave good predictions of experimental concentric torque-time curve with 10–14% error; well the prediction errors would go twice as large with generic muscle properties parameters [6]. In our previous study of elbow flexor modeling in the hemiparetic subjects, we also revealed significantly smaller RMS errors between the predicted and measured movement trajectories when using subject-specific dataset than that from applying cadaveric data from the literature [41].

Previous models using pure mathematical optimization or using outcome torque or force to inversely obtain musculotendon parameters have the limitations such as the following: only partial insight into relating the optimization outcome with explicit physically and biologically meaningful principles were provided; curve-fitting technique always used before the experimental torque-joint angle data to proceed and the fitting results might affect the effectiveness of the modeling to “unknown and unpredictable” values [42].

The optimal fascicle length is the muscle fascicle length at which a muscle can generate its maximum isometric force and it is generally assumed to be the fascicle length at which a muscle begins to develop passive force [27]. This parameter was found to be related to the amount of excursion of the muscle fiber over the force-length relationship [13, 30]. In this study, optimal fascicle length of each elbow extensor was obtained based on ultrasound measurement on muscle optimal angle. Our results showed the modeling data and ultrasound data were in the similar range which could be a cross-validation for the ultrasound measurement and the modeling calculation [28, 32, 36, 43, 44] (Table 2). In addition, the fascicle length from hemiparetic muscle seemed to have a larger deviation between ultrasound-measured data and modeling results in elbow extensors (Figure 5(b)), which might indicate the variation of the affected extensor properties in the isometric contraction. As seen in Figure 5, the estimation error may be due to the bad motor control ability (i.e., coactivation from elbow flexors) or the lower activation level since the estimation simply assumed the muscle was in fully activation (*a*(*t*) = 1). The extension simulation RMS increased at the extended joint positions (>80°) which may be caused by the underestimation of the moment arm at those positions.

### 4.3. Maximum Isometric Muscle Stress: A Modeling Output

Our results showed that there is no significant difference of maximum isometric muscle stress among the three elbow extensors in both healthy and stroke group which could be explained that the three-head-of-triceps brachii has similar force generation potential. As our expectation, we found that the maximum isometric muscle stress value from the hemiparetic group was significantly smaller than that found in the unimpaired group in extensor (Figure 6). This finding is in line with the study by Feng and coworkers [7]. The difference of the muscles stress between persons after stroke and healthy people could be explained in biomechanical and/or neurological factor. Previously, other investigators found that differences in muscle stress between muscle groups might be due to differences in specific tension of different muscle fiber or motor unit populations [45]. That is, the fast twitch fibers may have higher muscle stress than those of the slow twitch fibers. Fiber composition could be very different between subjects and/or between muscles. Moreover, it has been found by Robinson and coworkers that immobilization would result in significant increase in the proportion of no-force units and a trend toward preferential reduction in type FF units [46]. Furthermore, there is evidence on fiber measurements in biopsied muscle that supports the idea that the type FF or FR motor units are atrophied in patients with hemiparesis [47]. This implies reduction in number of fast twitch fibers after stroke. On the other hand, hypertrophy of type S motor units has also been reported, which reflects increase in the number of slow twitch fibers. Moreover, Young and Mayer (1982) had found a unique class of motor units in long-term hemiparetic muscle-slow-contracting and fatigable, which is not present in normal muscle [48]. These all substantiate that the muscle stress can be very different between subjects and/or between muscles, and then they form the rationales to optimize muscle stress in the current study.

Another factor to explain the lower muscle stress from persons after stroke was that some hemiparetic subjects might not be able to fully activate their muscles when instructed. There is evidence that this deficit in muscle activation is mostly due to reduced neural drive from higher centers (i.e., motor cortex) secondary to the lesion [49, 50]. The results suggested that the hemiparetic subjects could suffer from muscle weakness, which might be biomechanical or/and neurological in nature. In addition, there is a limitation in the current study that the calculation of PCSA is from the scaling of literature data which results in underestimation of the muscle stress from stroke group. The physiologic cross-sectional area of a muscle or, in essence, the number of sarcomeres in parallel is related directly to the amount of tension that the muscle can produce and the paretic arm may have significantly greater intramuscular fat and connective tissue than the unaffected or control arm. Further dedicated ultrasound study should combine measurements on transverse direction for cross section area with longitudinal direction on fascicle length and pennation angle to improve the accuracy.

In summary, this study considered and measured elbow extensor architectural changes after the onset of stroke and these in vivo parameters were used to build subject-specific biomechanical model. The maximum isometric muscle stress of elbow extensors from persons after stroke was found to be lower than that from control subjects, which indicated the weakness of muscle force generation potential after stroke. The technique and results in this study may help to evaluate the functional improvement of the affected muscle after an intervention program and to enhance the understanding of the effects on the muscle architecture and model during rehabilitation treatment.

## Figures and Tables

**Table 1:** Clinical characteristics of the 5 hemiparetic subjects including Modified Ashworth Scale [24].

Subject	Age (y) and sex	Years after injury	Arm affected	Modified Ashworth Score [24]
LI	63 (F)	3	L	3
CO	52 (M)	4	L	2
CH	61 (M)	2	R	1
ZH	52 (F)	4	L	1+
WO	44 (M)	4	L	1

F, female; M, male; R, right; L, left.

**Table 2:** Comparison of muscle optimal lengths and tendon slack lengths of prime elbow extensors of healthy group found in the study (mean value, *n* = 5) and those reported in other literature.

	Muscle	Holzbaur et al., 2005	Langenderfer et al., 2004	Murray et al., 2000	Winters and Stark, 1988	Garner and Pandy, 2003	Current study
Muscle optimal length (cm)	MHT	11.4	14.5	NR	6	8.77^*∗*^	8.8
	LatHT	11.4	10.3	6.6–13.9	7	8.77	11.0
	LngHT	13.4	17.6	9.5–16.5	9	8.77	9.2

Tendon slack length (cm)	MHT	9.1	NR	NR	17	19.05^*∗*^	5.7
	LatHT	9.8	NR	18.7	19	19.05	11.4
	LngHT	14.3	NR	21.7	22	19.05	18.7

NR: not reported. ^*∗*^Combination of MHT, LatHT, and LngHT as triceps brachii in Garner and Pandy, 2003.
